# *Mycobacterium avium* Possesses Extracellular DNA that Contributes to Biofilm Formation, Structural Integrity, and Tolerance to Antibiotics

**DOI:** 10.1371/journal.pone.0128772

**Published:** 2015-05-26

**Authors:** Sasha J. Rose, Lmar M. Babrak, Luiz E. Bermudez

**Affiliations:** 1 Department of Biomedical Sciences, College of Veterinary Medicine, Oregon State University, Corvallis, Oregon, United States of America; 2 Department of Microbiology, College of Science, Oregon State University, Corvallis, Oregon, United States of America; Ghent University, BELGIUM

## Abstract

*Mycobacterium avium* subsp. *hominissuis* is an opportunistic pathogen that is associated with biofilm-related infections of the respiratory tract and is difficult to treat. In recent years, extracellular DNA (eDNA) has been found to be a major component of bacterial biofilms, including many pathogens involved in biofilm-associated infections. To date, eDNA has not been described as a component of mycobacterial biofilms. In this study, we identified and characterized eDNA in a high biofilm-producing strain of *Mycobacterium avium* subsp. *hominissuis* (MAH). In addition, we surveyed for presence of eDNA in various MAH strains and other nontuberculous mycobacteria. Biofilms of MAH A5 (high biofilm-producing strain) and MAH 104 (reference strain) were established at 22°C and 37°C on abiotic surfaces. Acellular biofilm matrix and supernatant from MAH A5 7 day-old biofilms both possess abundant eDNA, however very little eDNA was found in MAH 104 biofilms. A survey of MAH clinical isolates and other clinically relevant nontuberculous mycobacterial species revealed many species and strains that also produce eDNA. RAPD analysis demonstrated that eDNA resembles genomic DNA. Treatment with DNase I reduced the biomass of MAH A5 biofilms when added upon biofilm formation or to an already established biofilm both on abiotic surfaces and on top of human pharyngeal epithelial cells. Furthermore, co-treatment of an established biofilm with DNase 1 and either moxifloxacin or clarithromycin significantly increased the susceptibility of the bacteria within the biofilm to these clinically used antimicrobials. Collectively, our results describe an additional matrix component of mycobacterial biofilms and a potential new target to help treat biofilm-associated nontuberculous mycobacterial infections.

## Introduction

*Mycobacterium avium* subsp. *hominissuis* (MAH) is an opportunistic human pathogen that typically infects individuals with underlying health conditions, such as AIDS, chronic obstructive pulmonary disease, and cystic fibrosis. Pulmonary infections with MAH and other nontuberculous mycobacteria are also increasing in incidence in patients without underlying conditions [1]. MAH is ubiquitous in the environment and is commonly found in potable water systems, presumably persisting in biofilms [2–4]. Studies have linked potable water reservoirs of MAH directly to infection in patients [5–7]. Previous work has suggested that the ability to form biofilm is associated with the efficiency to establish lung disease in mice [8]. Furthermore, we recently reported that MAH biofilm formed *in vitro* induces rapid, atypical TNF-α-dependent apoptosis of phagocytes exposed to the biofilm, which could explain how MAH biofilms formed *in vivo* establish and persist without host clearance [9]. This report additionally found that UV-sterilized biofilm was just as stimulatory to macrophages as non-sterilized live biofilm, suggesting that an acellular component of the biofilm matrix could be responsible for this rapid cell death upon interaction.

Little is currently known about the constituents of the extracellular polymeric substance (EPS) composing the matrix of *M*. *avium* biofilms. In most bacterial species, a major component of EPS are exopolysaccharides, however mycobacteria do not produce them, and lack the genes necessary for synthesis [10]. Studies have primarily found lipid EPS components of mycobacterial biofilms including free mycolic acids in *M*. *tuberculosis* and *M*. *smegmatis* [11, 12], glycopeptidolipids in *M*. *avium*, *M*. *abscessus*, and *M*. *smegmatis* [13–16], mycolyl-diacylglycerols in *M*. *smegmatis* [17], lipooligosaccharides in *M*. *marinum* [18], and lipopeptides in *M*. *avium* subsp. *paratuberculosis* [19].

In addition to exopolysaccharides, another important component of the EPS in bacterial biofilms is extracellular DNA (eDNA). It was first discovered in *Pseudomonas aeruginosa* biofilms by Whitchurch *et al*. in 2002 [20]. Since this report, eDNA has been described in many gram negative [21–25] and gram positive [26–32] bacteria. eDNA in these biofilms contribute to surface attachment and colonization [24–26, 28], biofilm recruitment [25, 29], structural integrity [21, 23, 27, 30–32], nutrient acquisition [25, 33], and a source of DNA for horizontal gene transfer [22, 34]. eDNA has also been shown to protect biofilms against antibiotics, biocides, and chemicals [27, 35–37]. Additionally, it was recently determined that eDNA facilitates cellular aggregation within *P*. *aeruginosa*, *Streptococcus mutans*, and *Staphylococcoc epidermidis* biofilms [38, 39], and assists in spatial self-organization in expanding *P*. *aeruginosa* biofilms [40].

The source of eDNA in bacterial biofilms is a controversial and ongoing issue. In *Pseudomonas aeruginosa*, *Staphylococcus* spp., and *Enterococcus* spp., eDNA has been connected to cell lysis [29–31, 35, 41]. Mechanisms of cell lysis contributing to eDNA production have included autolysin proteins [29–31], pyocyanins leading to H_2_O_2_ production and lysis [41], and quorum sensing resulting in prophage-mediated lysis [35]. There has also been a growing body of research suggesting active export in the absence of cell lysis as an alternative source of eDNA. One report investigating eDNA in *Enterococcus faecalis* early biofilms found no evidence of cell lysis and moreover the eDNA producing cells had elevated membrane potential [42]. Another study recently described early competence genes involved with eDNA production in *Bacillus cereus*, which also suggests a defined mechanism and not a mass cell die off as a trigger [32]. Secreted membrane vesicles are another mechanism that has been demonstrated to export eDNA outside of bacteria within biofilms [43, 44]. Other groups investigating eDNA production in *Helicobacter pylori* and an aquatic bacterium strain F8 also suggest eDNA release to be independent of cell lysis [22, 44, 45].

Prior work with *M*. *avium* biofilms has highlighted their importance for environmental persistence and possibly infection [8, 46]. MAH A5, in particular, has shown to be a strain that produces very robust and resistant biofilms [46], which induce very atypical host responses when compared to their planktonic counterparts [9]. Currently, little is known about the physical makeup of the MAH biofilm and the mechanisms responsible for it. In this study, we observed and characterized eDNA in MAH strain A5, which is the first report to date of eDNA as a biofilm matrix component in pathogenic mycobacteria. We also identified other clinically relevant nontuberculous mycobacteria that possess eDNA as a biofilm matrix component.

## Materials and Methods

### Bacterial Strains and growth

*Mycobacterium avium* subsp. *hominissuis* (MAH) strains A5 and 104 were originally isolated from the blood of AIDS patients. MAH strains 3386, 3388, and 3393 as well as *M*. *intracellulare* strain 3387 were generous gifts from Barbara Brown-Elliott (University of Texas Health Science Center). MAH Cl-3, MAH Cl-4, *M*. *intracellulare* Cl-2, and *M*. *abscessus* subsp. *bolletii* 26 were kindly provided by Steven Holland (National Institutes of Health). *Mycobacterium chelonae* strain h1e2 and *M*. *marinum* Harvard were gifted from Michael Kent (Oregon State University). *M*. *abscessus* subsp. *abscessus* strain 19977 was obtained from the American Type Culture Collection (Manassas, VA). The other mycobacteria used are strains isolated by our laboratory. All mycobacteria were grown on Middlebrook 7H10 agar plates containing 10% oleic acid, albumin, dextrose, and catalase (OADC; Hardy Diagnostics, Santa Maria, CA). MAH, *M*. *intracellulare*, and *M*. *abscessus* strains were grown at 37°C. All other mycobacterial species were grown at 30°C.

### Host Cells

Human HEp-2 pharyngeal epithelial cells (CCL-23) were obtained from the American Type Culture Collection and cultured in RPMI-1640 media containing 2 mM L-glutamine and 25 mM HEPES (Cellgro, Manassas, VA) further supplemented with 10% heat inactivated fetal bovine serum (FBS, Gemini Bio-Products, Sacramento, CA) at 37°C with 5% CO_2_. Confluent monolayers of cells were removed using TrypLE (Life Technologies, Carlsbad, CA), washed, resuspended in fresh media, counted with a hemocytometer, and seeded into 96-well plates for later biofilm formation experiments.

### Biofilm formation

Static biofilms were used in this study, formed similarly to previous reports [9, 46], with minor modifications. Briefly, log-phase bacteria were transferred from 7H10 agar into Hank’s Balanced Salt Solution (HBSS; Cellgro) to create a mid-10^8^ bacteria/ml suspension, using visual turbidity. This suspension was left alone for 10 minutes to allow clumped bacteria to sediment. The top half of the suspension was withdrawn, and adjusted to 3 x 10^8^ bacteria per ml, using a McFarland #1 standard as a reference. This suspension was transferred into an appropriate polystyrene vessel (listed for each experiment) and incubated at 22°C, 30°C, or 37°C for the time point indicated by the specific experiment.

### Scanning Electron Microscopy

Biofilms visualized via scanning microscopy were prepared as described above and processed for microscopy as previously described [42]. Briefly, biofilms were prepared on top of round coverslips in the bottom of wells of a 24-well culture plate. At 7 days of biofilm maturation, the supernatant was removed and the coverslips were gently rinsed three times with HBSS and then blocked with 2% bovine serum albumen. Samples were labeled with a primary mouse antibody against dsDNA (Abcam, Cambridge, MA) and then after three washes, a donkey anti-mouse secondary antibody conjugated to 12 nm colloidal gold particles (Abcam). Labeled samples were fixed in a solution of 150 mM sodium cacodylate, 2% formaldehyde, 2% glutaraldehyde, 4% sucrose, and 0.15% Alcian blue 8GX (Sigma Aldrich, St. Louis, MO) at room temperature for 22 hours. Samples were then dried in graded ethanol concentrations (25%, 50%, 70%, 85%, 95%, 95%, 100%, 100%), further dried in a CO_2_ critical point dryer, and sputter coated with 10–15nm of gold palladium. Electron micrographs were captured using a FEI Quant 600F FE scanning electron microscope at the Oregon State University Electron Microscopy Facility.

### Biofilm matrix and supernatant preparation

Seven day-old biofilms grown in 75 cm^2^ flasks were gently removed using a sterile swab, collected, and centrifuged at 2,500 x *g* for 15 minutes to pellet the biomass. The biofilm supernatant was poured off, collected, and filtered through a 0.2μm syringe filter. The supernatant was then concentrated 30x using 30 kDa pore size centrifugal filter (Pall Corporation, Port Washington, NY). The remaining biomass pellet after supernatant removal was resuspended into 1 ml of sterile deionized H_2_O and physically agitated in a Mini-Beadbeater-1 (Biospec Products, Bartlesville, OK) at 4,800 oscillations per minute for 1 minute without beads, to not lyse the bacteria. Samples were centrifuged 12,000 x *g* for 5 minutes to pellet the bacteria, but keep the solubilized matrix in suspension. The liquid was withdrawn, and filtered through a 0.2μm syringe filter to sterilize the extracted matrix.

### Quantification of eDNA

25 μl of biofilm matrix or supernatant was added into 4 replicate wells of a black walled 96-well plate and then 25 μl of 6 μM propidium iodide (in deionized H_2_O) was added to each well. The plate was briefly vortexed, incubated at room temperature in the dark for 15 minutes, and then fluorescently quantified using an Infinite F200 microplate reader (Tecan, Männedorf, Switzerland) with an excitation filter at 535 nm and an emission filter at 620 nm. As a background control, deionized H_2_O was mixed with the propidium iodide to attain the background fluorescence of the stain. The mean of the background value for each plate was subtracted from the sample values obtained, thus fluorescent signal reported is directly correlated to presence of DNA.

### RAPD PCR, cloning, and sequencing of eDNA

Genomic DNA (gDNA) from log-phase MAH A5 was extracted using the DNeasy Blood and tissue kit (Qiagen, Venlo, Netherlands). eDNA was extracted in the matrix described above and further purified using a DNA Clean & Concentrator kit (Zymo Research, Irvine, CA). RAPD (Randomly Amplified Polymorphic DNA) PCR was conducted on eDNA and gDNA using previously published random primers that worked successfully with *M*. *avium*: primer set 1, 5’-AACGCGCAAC-3’ [47]; primer set 2, 5’-AAGAGCCCGT-3’ [47]; and primer set 3, 5’-AACGGTGACC-3’ [48]. The reaction was as follows: 92°C for 1 minute, 35°C for 1 minute, and 72°C for 1 minute for 45 cycles followed by a final extension period of 7 minutes. RAPD products were separated and visualized using 2% agarose gel electrophoresis containing 0.5 μg/ml of ethidium bromide in the gel. For cloning and sequencing of random eDNA fragments, purified eDNA was double-digested for 2 hours at 37°C with BamHI and BglII, PCR purified (Qiagen), ligated into a similarly digested pMV261 mycobacterial shuttle vector, and then electroporated into *Escherichia coli* DH10B (Life Technologies). Inserts from 7 random transformants were sequenced at the Center for Genome Research and Biocomputing at Oregon State University. BLAST from NCBI was used to identify the returned sequences and the *M*. *avium* 104 annotated genome (NCBI Reference Sequence: NC_008595.1) was used as a reference genome for gene identifications.

### DNase I experiments

Recombinant DNase I (Roche, Penzberg, Germany) was used at 100 Kunitz units per ml in all experiments to analyze biofilm prevention, removal, and tolerance. These experiments were carried out in 96-well polystyrene plates. To assess the effect of DNase on biofilm formation, biofilms were formed, as described earlier, with the addition of 10% v/v 10x DNase buffer (400 mM Tris-HCl, 100 mM NaCl, 60mM MgCl2, 10 mM CaCl2; pH 7.9) into the inoculum with or without DNase. After 1 day of biofilm formation, supernatants were withdrawn, serially diluted, and plated to attain CFU of non-adherent bacteria.

For established biofilm removal experiments, biofilms were formed in HBSS as described earlier on abiotic surfaces and for the HEp-2 experiments, the bacterial inoculum was created in RPMI media instead of HBSS and the plates were incubated only at 37°C with 5% CO_2_. At 7 days of biofilm formation the supernatant was gently removed and replaced with 1x DNase buffer in HBSS with or without DNase for 2 hours at 37°C (even for 22°C biofilms). For both experiments, supernatant was withdrawn, serially diluted, and plated to determine CFU of removed bacteria.

For antibiotic tolerance experiments, stock solutions of moxifloxacin (Sigma) and clarithromycin (TCI, Tokyo, Japan) were made at 20 mg/ml in dH_2_O and acetone, respectively. Moxifloxacin and clarithromycin were used at 16 μg/ml for experiments. Biofilms were formed in HBSS for 7 days, and then supernatants were gently removed and replaced with 1x DNase buffer with antibiotic with or without DNase and incubated at 37°C for 48 hours (even for 22°C biofilms). This experiment was unique from the others, in that after treatment, supernatant was not removed from the wells; instead, the entire population of bacteria (both unadhered and adhered) were mixed, diluted, and plated for CFU. Wells were mixed well by pipetting 50x, diluted, and plated to attain CFU of surviving bacteria.

### Statistical Analysis

Statistical comparisons were made using an unpaired homoscedastic t-test. Definitions of statistical significance, where shown in figures by asterisks, are described in the corresponding Figure caption. Microsoft Excel and GraphPad Prism were used for statistics and graphical outputs, respectively.

## Results

### Extracellular DNA is abundant in the MAH A5 biofilm matrix and supernatant

**Fig 1:**
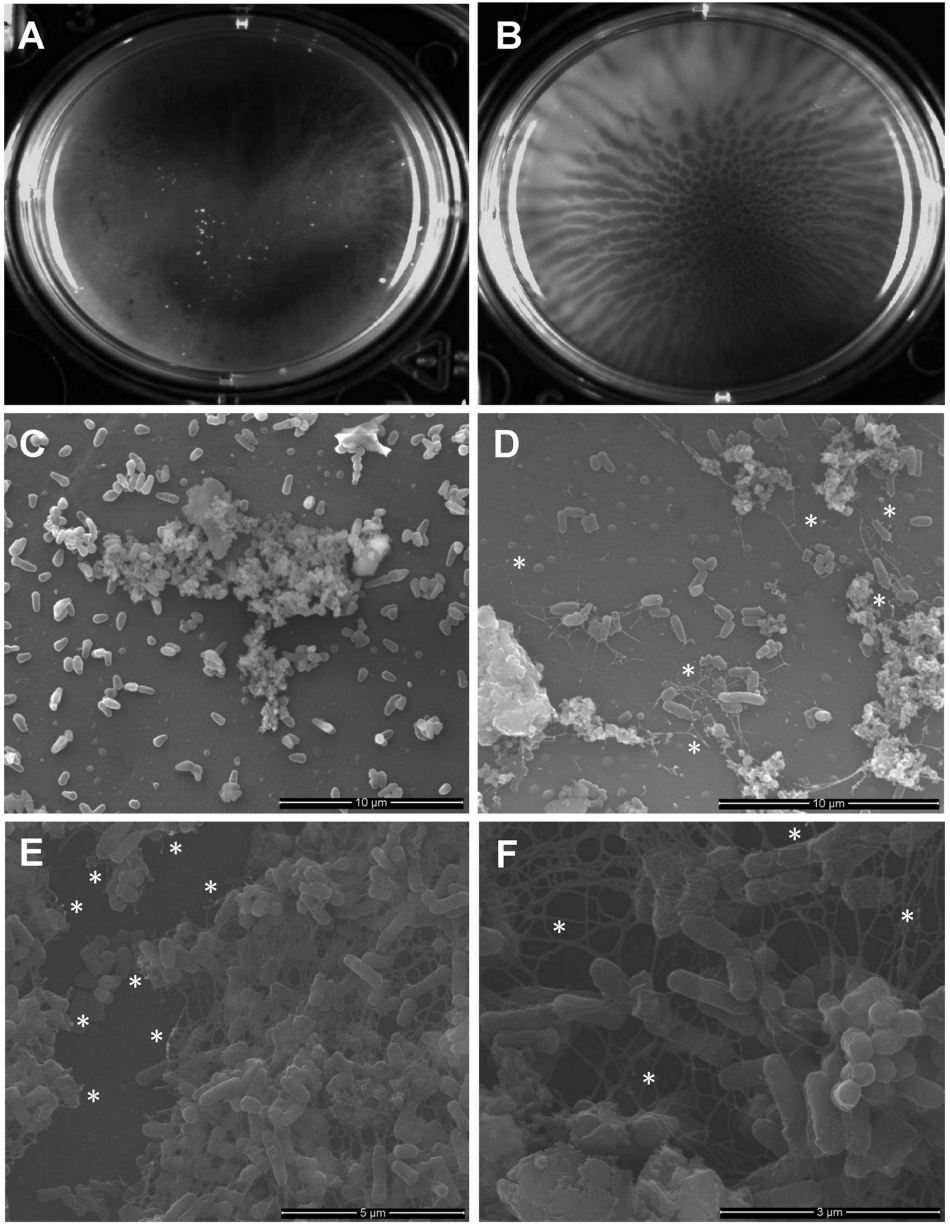
Gross morphology and scanning electron microscopy of MAH 104 and A5 7 day-old biofilms. Log-phase bacteria were resuspended into HBSS and were incubated statically for 7 days at room temperature to allow biofilm formation on an abiotic surface at the surface-liquid interface. (A and B) Gross morphology of a biofilm formed in a 6-well polystyrene plate. (C–F) Biofilms formed on top of coverslips were fixed, labeled with an anti-dsDNA primary antibody, labeled with a secondary antibody conjugated to gold particles, and visualized with scanning electron microscopy. MAH 104 (C) and MAH A5 (D–F) both have biofilm matrix, but the eDNA is only observed in MAH A5 (fibrous structures). Gold particles bound to eDNA, appearing as white spots, were only visualized in MAH A5 biofilms and were localized to the filamentous structures. Asterisks are placed in the micrographs to demonstrate locations of immunogold particles.

Despite the growing body of research suggesting eDNA as a major component of microbial biofilms, there have been no reports to date of eDNA as a constituent of the mycobacterial biofilm matrix. We first assessed if MAH 104 and MAH A5 possess eDNA when grown in biofilms *in vitro*. Previous work has characterized MAH A5 as a high biofilm producing strain, when compared to other MAH clinical isolates [46] and MAH 104 is the genome-sequenced reference strain. Macroscopically, surface-attached biofilms from MAH 104 (Fig 1A) and MAH A5 (Fig 1B) have differing phenotypes. Additionally, MAH A5 has a distinct wrinkling pattern, which is consistent with other reports of eDNA-producing biofilms [49]. To visualize if eDNA was present in these biofilms, scanning electron microscopy with dsDNA-specific immunogold labeling was carried out on 7 day MAH A5 and 104 biofilms formed in HBSS on abiotic surfaces at 22°C (Fig 1C–1F). A network of fibrous structures are seen in the MAH A5 biofilm with immunogold particles being localized on these structures (Fig 1D–1F), which is consistent with eDNA recently visualized in *Enterococcus* biofilms [42]. Though the biofilm as not as intact after preparation, biofilm matrix was seen in the MAH 104 biofilm, but it was not fibrous and did not contain any bound immunogold particles (Fig 1C).

**Fig 2:**
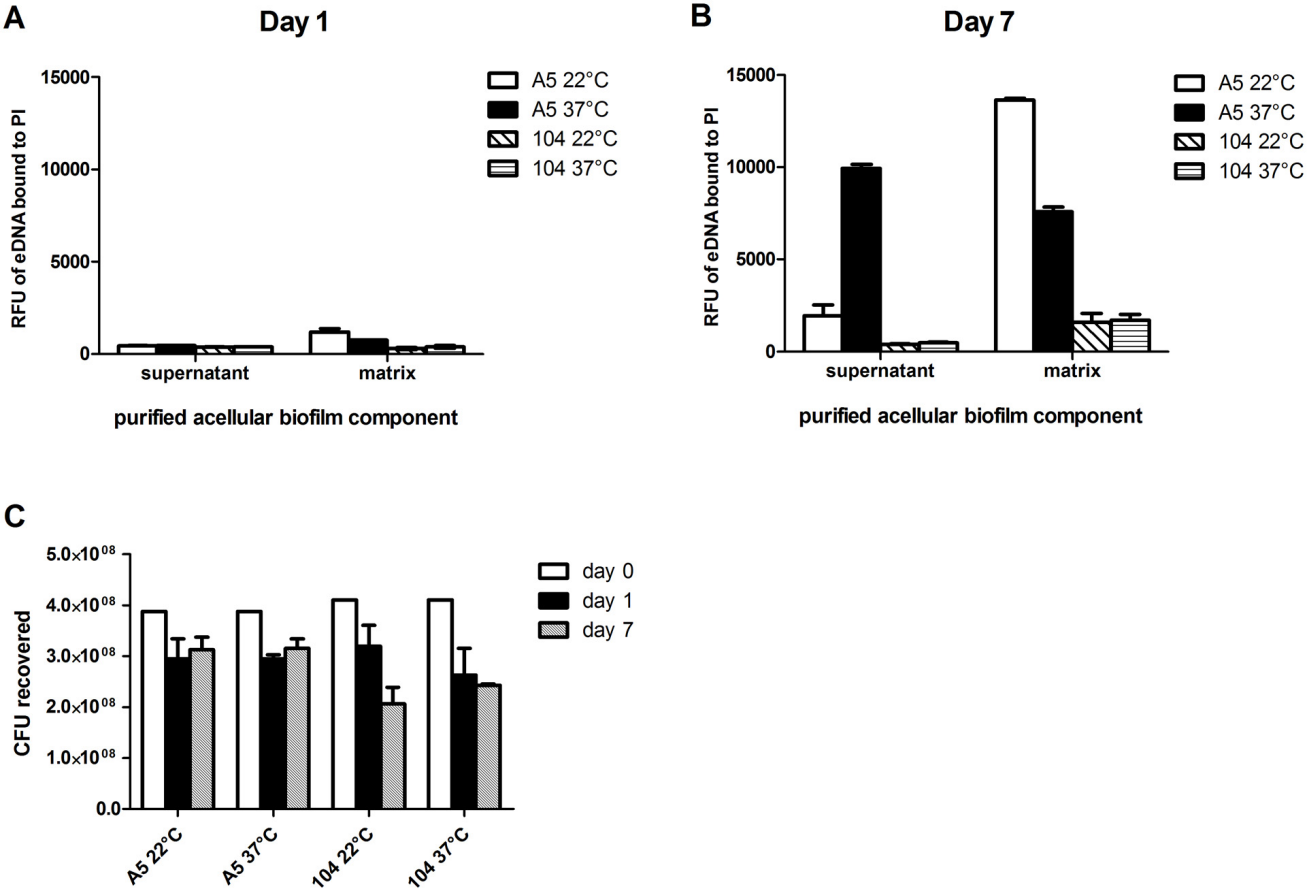
Quantitative assessment of eDNA and CFU in MAH A5 and 104 biofilms. MAH A5 and 104 static biofilms were formed in 75 cm^2^ flasks in HBSS at both 22°C and 37°C to represent environmental and host-associated biofilms, respectively. (A and B) After 1 and 7 days of biofilm formation, acellular matrix and supernatant was collected from the biofilms and quantitatively assessed for eDNA using propidium iodide. Bars represent the mean of three biological replicates ± the standard error of the mean (day 7) and the mean of two biological replicates ± the standard error of the mean (day 1). RFU: Relative fluorescence units. PI: Propidium iodide. (C) As biofilms formed over 7 days, wells were mixed thoroughly to remove adhered bacteria and samples were processed for CFU. Day 0 represents the CFU of the inoculum prior to seeding wells for biofilm production. Bars represent the average of three wells of biofilm processed independently ± the standard deviation. The data shown is representative of three biological replicates.

Due to the observation suggesting eDNA in MAH A5 biofilms, next we quantitatively assessed eDNA in acellular biofilm components. Biofilms were statically formed on abiotic surfaces both at 22°C and 37°C, to represent environmental and human host associated biofilms, respectively. After 1 and 7 days of biofilm formation, supernatant was collected and concentrated, and the water soluble biofilm matrix was extracted. The acellular matrix and supernatant were quantified with a fluorescent plate reader after mixing with propidium iodide, a stain specific for DNA. When comparing day 1 to day 7, there is a significant increase in eDNA production over the time course (Fig 2A and 2B). At day 7 of biofilm formation, MAH A5 possesses abundant eDNA in its acellular matrix and supernatant formed at both 22°C and 37°C, whereas MAH 104 produced significantly less eDNA at both temperatures (Fig 2B). Taken together, these data suggest that eDNA is a major component of MAH A5 biofilms.

To assess how the number of viable bacteria in the biofilm correlated with eDNA production, CFU of MAH A5 and 104 were enumerated over the course of biofilm formation (Fig 2C). There is a slight drop in CFU for both strains between the original inoculum and days 1 and 7, but the drop is larger in MAH 104, which produces significantly less eDNA. Furthermore, eDNA measured from day 1 MAH A5 biofilms is substantially less than day 7 (Fig 2A and 2B), which indicates that the minor drop in CFU does not correlate with presence of eDNA, since day 1 and 7 MAH A5 CFU recovered are similar (Fig 2C).

### eDNA is found among MAH and other nontuberculous mycobacteria

**Fig 3:**
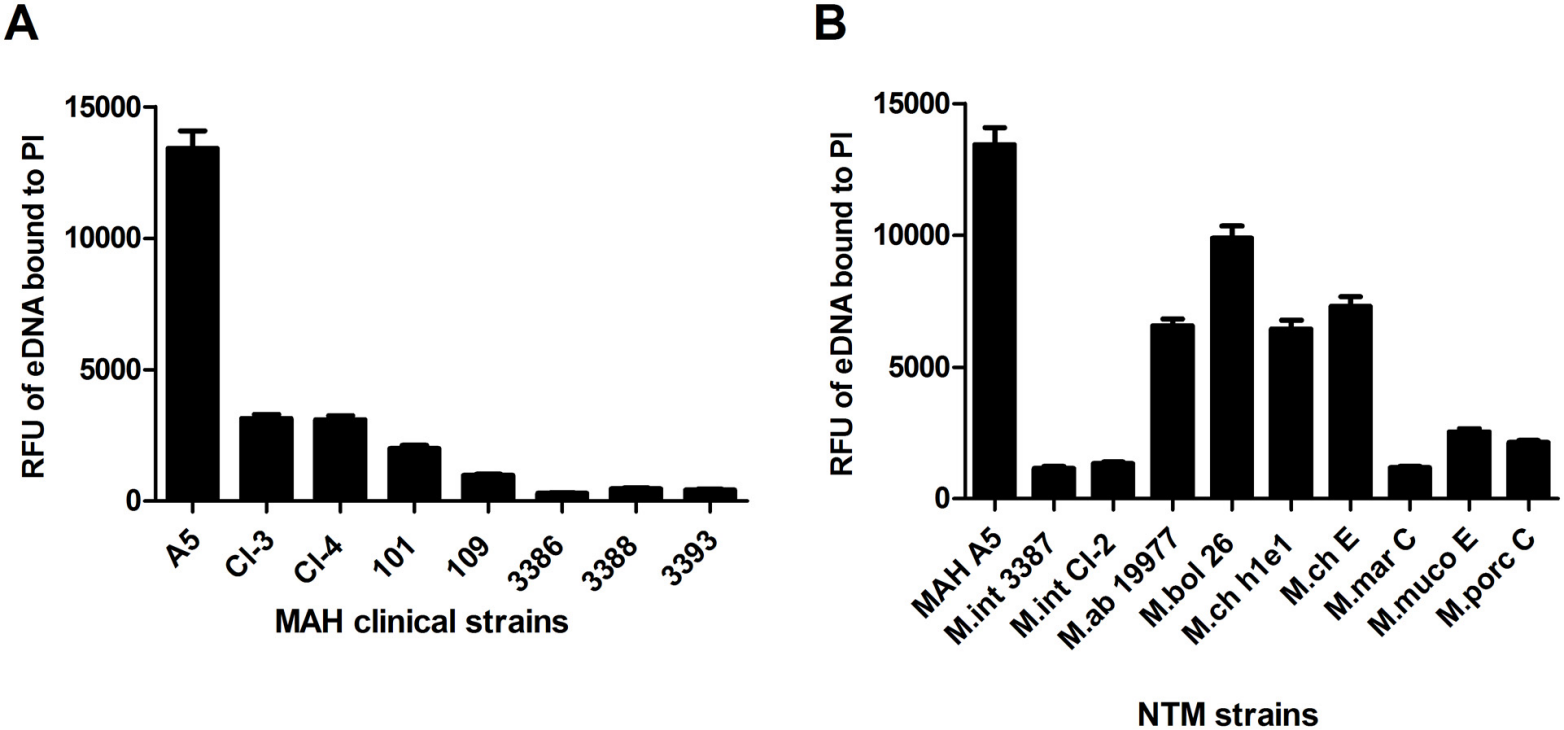
Survey of eDNA in other MAH strains and nontuberculous mycobacteria. To assess if eDNA was widespread throughout mycobacteria and not an artifact of MAH A5, biofilms were formed in (A) other *M*. *avium* strains and (B) other nontuberculous mycobacteria. Biofilms were formed in 75 cm^2^ flasks and after 7 days of formation at 30°C, acellular biofilm matrix was extracted and quantified for eDNA. Abbreviations used: M.int = *M*. *intracellulare*; M.ab = *M*. *abscessus* subsp. *abscessus*; M.bol = *M*. *abscessus* subsp. *bolletii*; M.ch = *M*. *chelonae*; M.mar = *M*. *marinum*; M.muco = *M*. *mucogenicum*; M.porc = *M*. *porcinum*; E = environmental isolate; C = clinical isolate; RFU = relative fluorescence units; PI = propidium iodide.

To survey if eDNA production was specific to MAH A5, or more widespread among MAH, we extracted acellular biofilm matrix from other MAH clinical isolates and quantified eDNA. Though MAH A5 possesses more eDNA in its biofilm matrix than other MAH strains tested, the data shows that other MAH strains do produce eDNA (Fig 3A). Due to the known heterogeneity of the species, it is not unexpected that the eDNA concentrations are variable among strains. We further expanded the assessment of eDNA production beyond MAH to other clinically relevant, biofilm forming nontuberculous mycobacteria (Fig 3B). Similar to *M*. *avium*, eDNA levels are variable in different species, but it does appear that *M*. *abscessus* and *M*. *chelonae*, regardless of strain, produce considerably more eDNA than the other mycobacterial species tested. Since MAH A5 produces the most eDNA of the mycobacterial species tested, it being characterized previously as a high biofilm producer, and our interest in its pathogenesis, we chose to concentrate on MAH A5 for the rest of the study.

### Characterization of eDNA in MAH A5

**Fig 4:**
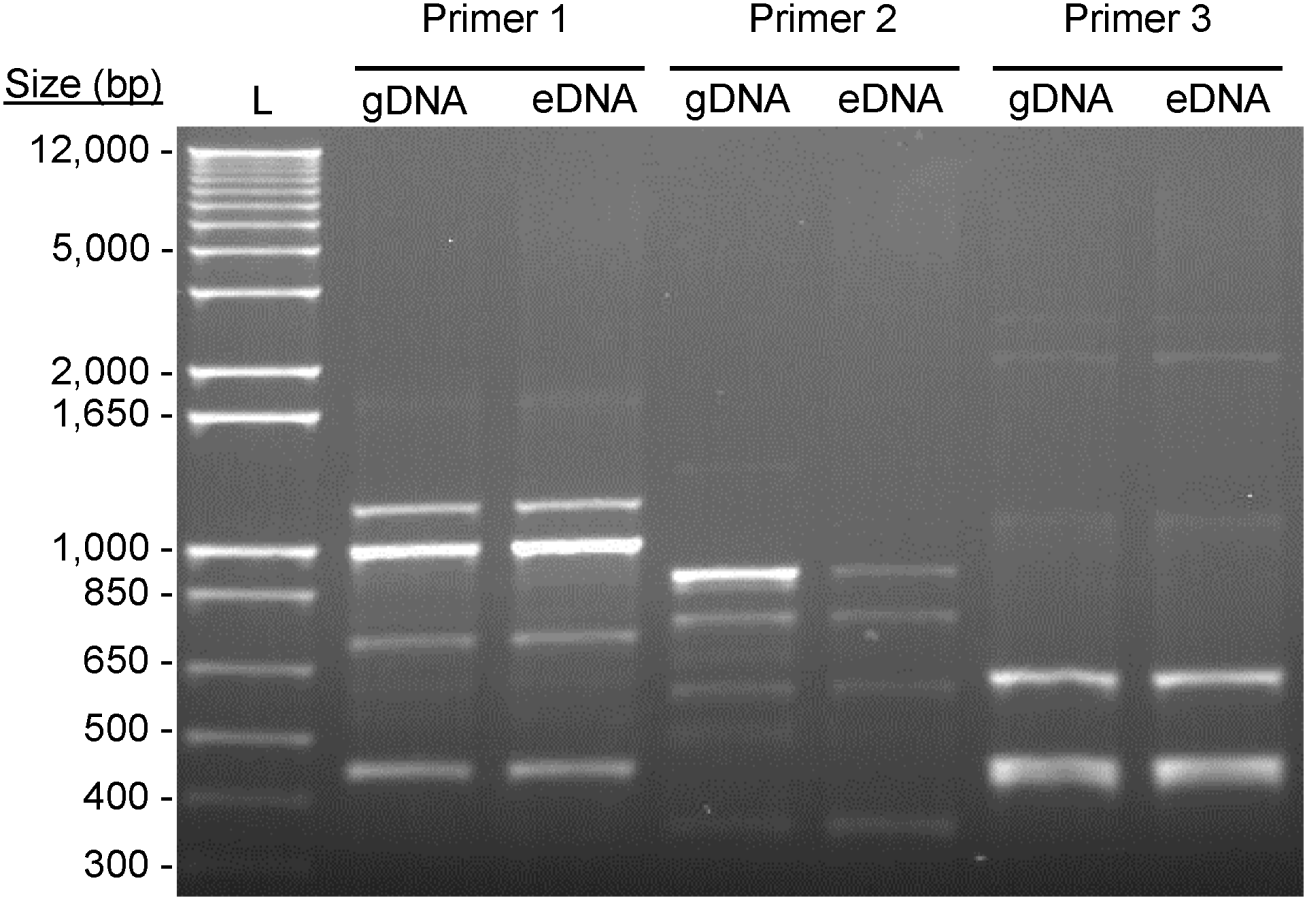
Characterization of eDNA in the MAH A5 biofilm. To compare the similarities of eDNA with genomic DNA (gDNA), side-by-side fingerprinting was done. eDNA extracted from the A5 biofilm matrix was purified and gDNA was extracted from log-phase A5 using a commercial kit. RAPD PCR was conducted from these DNA templates using three sets of random primers that have already been confirmed to work with *M*. *avium*.

To investigate if eDNA found in the biofilm matrix is genomic in origin or an eDNA-specific sequence, it was directly compared to genomic DNA (gDNA) from log-phase MAH A5. Random amplified Polymorphic DNA (RAPD) PCR was used to gauge the similarity between extracted eDNA and gDNA with the rationale that a specific secreted sequence of DNA (not containing the entire genome) will produce a RAPD fingerprint distinct from genomic DNA. The RAPD fingerprint between eDNA and gDNA using three proven RAPD primers were all indistinguishable (Fig 4), suggesting eDNA is genomic in origin. To corroborate this, extracted eDNA was digested with BamHI and BglII, ligated into a similarly digested pMV261 mycobacterial-*E*. *coli* shuttle vector, electroporated into *E*. *coli*, and plasmid insertions from 7 transformants were sequenced. The returned sequences were distributed randomly throughout the MAH genome (Table 1), further confirming that the eDNA is genomic in origin and not a particular sequence unique to eDNA.

**Table 1:** Sequence results from 7 random pieces of eDNA that were extracted from the MAH A5 biofilm matrix, digested, purified, cloned into a shuttle plasmid, and sequenced.

Sample ID	Gene ID*	Gene description
1	MAV_0734	Hypothetical protein
	MAV_0735	Phosphoribosylformylglycinamidine synthase, PurS
	MAV_0736	Phosphoribosylformylgylcinamidine synthase I
2	MAV_2472	Isochorismatase family protein
	MAV_2473	Nuclear transport factor 2 (NTF2) domain family
3	MAV_5173	Putative acyl-CoA dehydrogenase
	MAV_5174	ZbpA protein
4	MAV_4973	MmpL11 protein
5	MAV_4859	Trans-aconitate 2-methyltransferase
6	MAV_4893	Hypothetical protein
	MAV_4892	Nitrite transporter
7	MAV_4541	Cyclopropane-fatty-acyl-phospholipid synthase
	MAV_4542	Exodeoxylribonuclease V, gamma subunit

* The MAH 104 annotated genome (NCBI Reference Sequence: NC_008595.1) was used as a reference genome for gene identifications.

### eDNA has a structural role in MAH A5 biofilms

**Fig 5:**
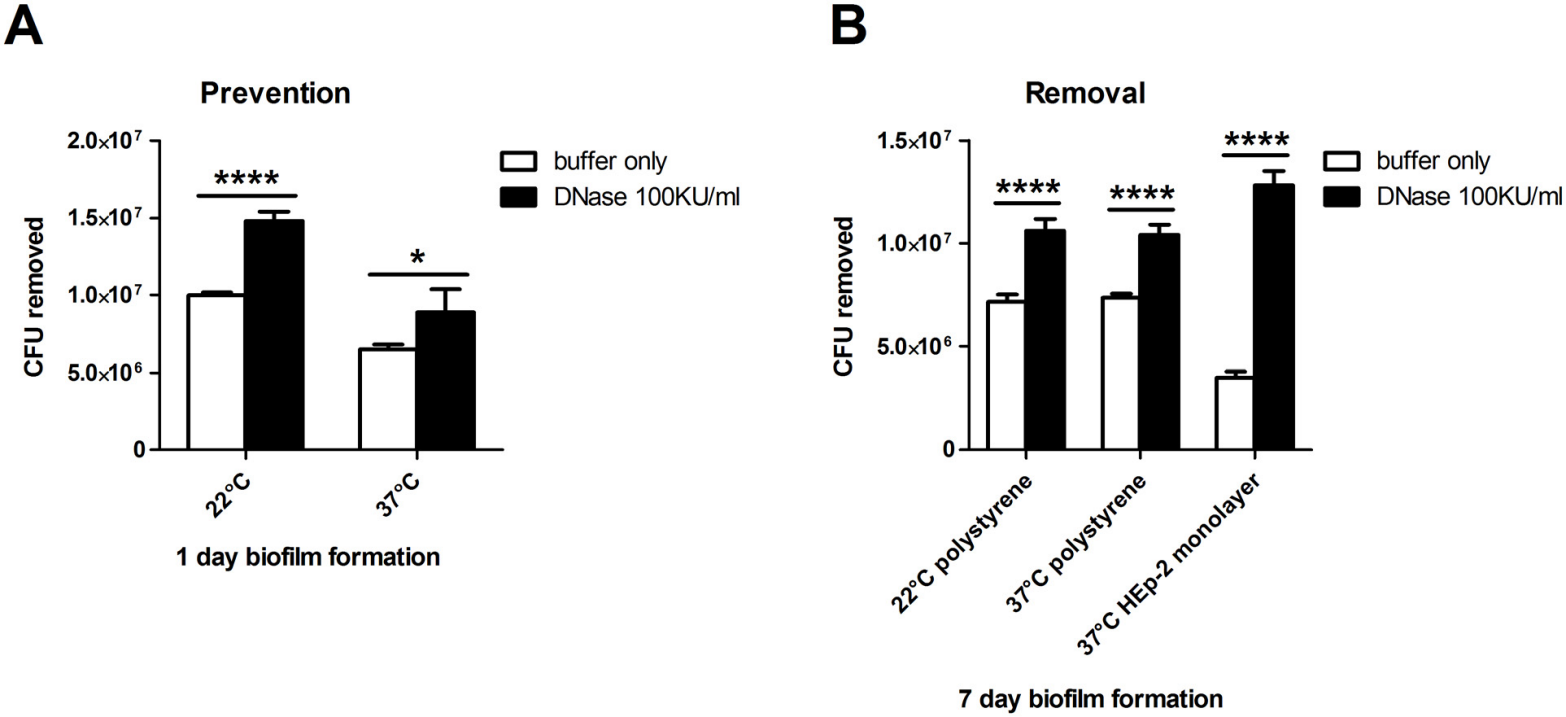
Effect of DNase treatment on preventing and removing MAH A5 biofilms. (A) Biofilms inoculums were made in HBSS supplemented with 1x DNase buffer ± 100 Kunitz units per ml of recombinant DNase I. After 1 day of static biofilm formation at both 22°C and 37°C, supernatants were removed to enumerate the CFU of non-adhered bacteria. (B) For abiotic surface removal, biofilms were formed in 96-well plates in HBSS for 7 days at both 22°C and 37°C. For HEp-2 surface removal, biofilms were formed in RPMI media supplemented with 10% FBS on top of already established HEp-2 monolayers in 96-well plates. At 7 days, supernatants were gently removed and replaced with HBSS supplemented with 1x DNase buffer ± 100 Kunitz units per ml of DNase. Biofilms were incubated for an additional 2 hours and then supernatants removed to enumerate CFU of detached bacteria. For both A and B, bars represent the average of 4 wells of biofilm processed independently ± standard deviation. Data sown are representative of three biological replicates. Statistical comparisons: * *P* < 0.05; **** *P* < 0.0001.

Previous work describing MAH A5 as a biofilm overproducer [46], the robustness of the biofilm (Fig 1B), and the fibrous eDNA matrix (Fig 1D–1F) led us to hypothesize that eDNA could participate in a structural role in the A5 biofilm. To investigate this, DNase I was used to prevent biofilm formation as well as to destabilize established biofilms. After 1 day of biofilm formation with the addition of DNase buffer with or without DNase I to the biofilm inoculum, the supernatants were removed to quantify the amount of unadhered bacteria. The combination of DNase buffer and DNase significantly inhibited the establishment of MAH biofilms after 1 day of formation, when compared to the addition of DNase buffer by itself (Fig 5A). This effect was observed in biofilms formed both at 22°C (48% more bacteria removed) and 37°C (37% more bacteria removed).

To assess the structural contribution of eDNA to established A5 biofilms, DNase was added after 7 days of biofilm formation by gently removing the biofilm supernatant and replacing with DNase buffer with or without DNase. DNase buffer with DNase was significantly more effective at removing MAH A5 established biofilms than DNase buffer by itself (48% and 41% more bacteria removed from 22°C and 37°C biofilms, respectively) after a 2 hour digestion period (Fig 5B). To further determine the structural contribution of eDNA to host-associated biofilms, MAH A5 biofilms were formed on top of a HEp-2 pharyngeal cell monolayer for 7 days and then subjected to a 2 hour DNase digestion. Similar to biofilms formed on top of abiotic surfaces, DNase also significantly removed more bacteria from established biofilms on top of HEP-2 cells, when compared with DNase buffer by itself (Fig 5B). The effect was more pronounced in these experiments (268% more bacteria removed), which suggests that the biofilms formed on top of host cells could be different from biofilms formed on abiotic surfaces. Collectively, these data demonstrate that eDNA plays a considerable structural role in MAH A5 biofilms.

### eDNA aids in tolerance of the MAH A5 biofilm to antibiotic treatment

**Fig 6:**
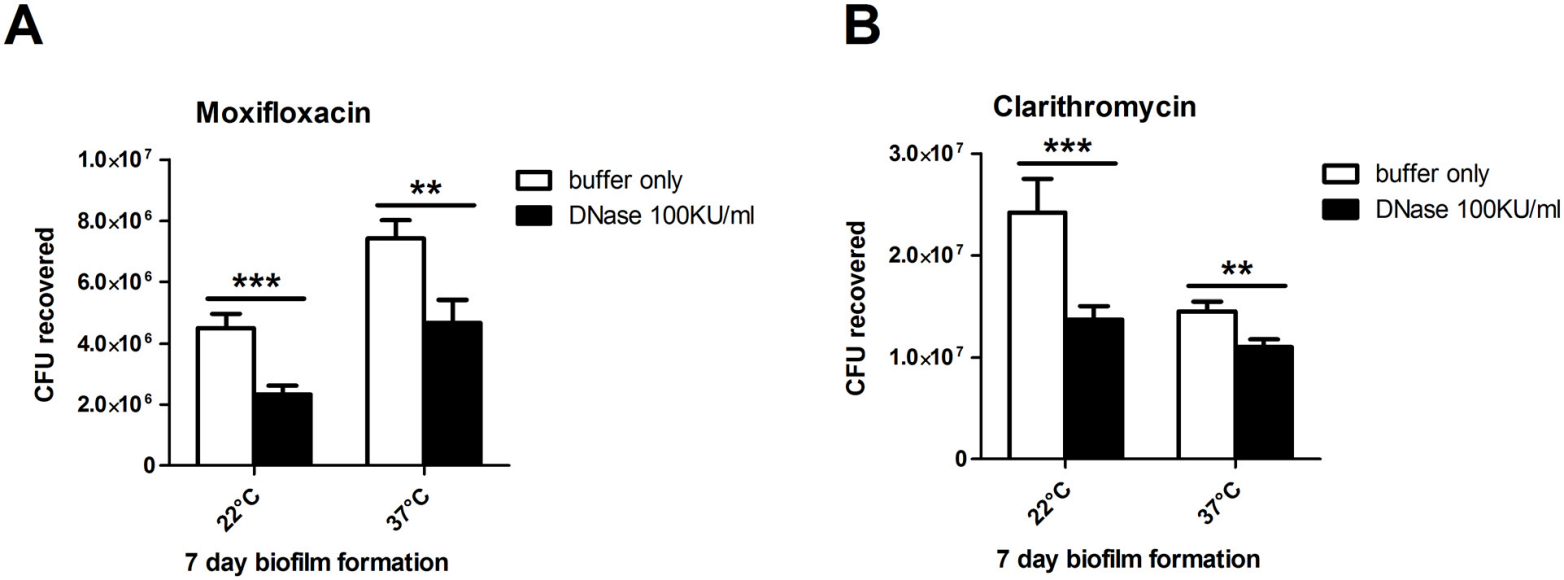
Effect of DNase co-treatment with antimicrobials on the MAH A5 biofilm. Biofilms were formed in HBSS in 96-well plates and incubated at 22°C and 37°C for 7 days. Supernatants were gently removed and replaced with HBSS containing 16 μg/ml of (A) moxifloxacin or (B) clarithromycin further supplemented 1x DNase buffer ± 100 Kunitz units per ml of recombinant DNase. All biofilms (including 22°C) were incubated for 48 hours at 37°C. Wells were mixed 50 times via pipetting to remove attached bacteria and samples were diluted and plated to obtain total viable bacteria. For both A and B, bars represent the average of 4 wells of biofilm processed independently ± standard deviation. Data shown are representative of three biological replicates. Statistical comparisons: ** *P* < 0.01; *** *P* < 0.001.

MAH biofilms have been shown to be more resistant to antimicrobials than their planktonic counterpart [50]. Since the DNase prevention and removal work demonstrated the structural role of eDNA in these biofilms (Fig 5), we further assessed if co-treatment of DNase with an antimicrobial would reduce the tolerance of the biofilm. Moxifloxacin and clarithromycin, which are both used clinically against MAH [1], were used in these experiments. Biofilms were formed for 7 days on an abiotic surface, and then the supernatant was removed and replaced with 16 μg per ml of moxifloxacin or clarithromycin in DNase buffer with or without DNase and incubated at 37°C for 48 hours. Without washing away or removing any bacteria first, entire biofilm wells (containing both unadhered and adhered bacteria) were resuspended and plated to attain CFU of surviving bacteria. Bacteria in the biofilms co-treated with DNase were significantly more sensitive to both moxifloxacin and clarithromycin than bacteria in biofilms that were co-treated with DNase buffer only (Fig 6A and 6B, respectively). For moxifloxacin, there were 48% less viable bacteria at 22°C and 37% less at 37°C. For clarithromycin, there were 43% less at 22°C and 24% less at 37°C. Taken together, these data show that eDNA in the biofilm contributes to the tolerance of MAH to commonly used antibiotics for MAH treatment.

## Discussion

Extracellular DNA (eDNA) has been described in many bacterial biofilms in recent years, but until this point, has not been identified in mycobacterial biofilms. We found and characterized eDNA in a previously reported high biofilm forming strain of *Mycobacterium avium* subsp. *hominissuis*, MAH A5. Furthermore, we report that eDNA in mycobacterial biofilms is more widespread and not just unique to MAH A5, even though of the species and strains tested, it appears to be the largest producer. The eDNA in MAH A5 biofilms appears to be of genomic origin, aids in the structural integrity, and contributes to antimicrobial tolerance.

The source of eDNA, whether it be a result of cell lysis or is actively secreted, is a controversial subject. Multiple reports have described lysis as the mechanism [29–31, 35, 41], but in recent years, growing evidence is suggesting that in some organisms, eDNA production could result from non-lytic mechanisms [22, 32, 42–45]. We assessed if the numbers of viable bacteria correlated with was responsible for eDNA production in MAH A5 by tracking CFU of MAH in the biofilm over the experimental time course. At day 1 and 7 there are similar minor reductions in the MAH A5 CFU from the initial inoculum, but this can be attributed to insufficient removal of all of the bacteria out of the microplate due to the lipid rich biofilm and visual confirmation of residue left in the wells. Interestingly, there was very little difference between CFU recovered between day 1 and day 7, even though the eDNA quantified increased dramatically between these two time points. Barnes *et al*. described lysed cells and subsequently released eDNA in electron micrographs and that in their early biofilm micrographs, lysed cells were infrequently encountered [42]. Our electron micrographs agree with this in that we largely observe intact bacilli within the A5 biofilm (Fig 1F). More research will be needed to answer if cell lysis contributes to MAH eDNA production, but our data presented gives preliminary evidence that other mechanisms could be contributing to eDNA production, such as membrane vesicle secretion [43, 44]. Secreted membrane vesicles have been found in mycobacteria, including *M*. *avium*, but eDNA in these vesicles has not been reported [51, 52].

When comparing the fibrous or strand-like eDNA structures observed in MAH A5 with other reports [42, 45], there are visual similarities. The fibrous eDNA network in the environmental strain F8 from Böckelmann *et al*. is very similar to our observations. The strands seen in Barnes *et al*. do not form a network as complex as ours or in Böckelmann *et al*., but these were early biofilms studied (2–8 hours), compared with ours (7 days) and Böckelmann *et al*. (4 days). We are confident that the fibrous matrix observed in the A5 biofilm is, at least partly, composed of eDNA, but there were limitations in the immunogold labeling. When comparing the MAH A5 biofilm with the *Enterococcus* biofilm in Barnes *et al*., there is reduced antibody labeling. It can be assumed that the matrix of early *Enterococcus* biofilms is less complex than of our 7 day MAH A5 biofilms, which could yield more accessible eDNA targets for antibody binding. It is plausible that the complex, lipid rich matrix of MAH A5 either making the eDNA inaccessible to the antibody, or that a component in the matrix is non-specifically binding the antibody and inactivating it. Interestingly, the labeling occurred most frequently at biofilm wrinkles or breaks, which there were possibly more exposed portions of eDNA.

It is important when studying environmental opportunistic pathogens to discern environmental mechanisms and processes from host-specific ones. A goal of this study was to investigate this newly found eDNA under conditions representing environmental and host-related biofilms, to help elucidate if eDNA production in *M*. *avium* is an environmental adaptation, a mechanism used for virulence, or possibly used for both. When quantifying eDNA from acellular A5 supernatant and matrix (Fig 2A), there were differences between biofilms grown at 22°C and 37°C (less eDNA in the matrix than 22°C but more in the supernatant). When analyzing the effect of DNase on MAH A5 biofilm structure and antimicrobial tolerance, there were consistently greater reductions at 22°C than at 37°C, but due to the differing matrix/supernatant eDNA quantifications between the temperatures, a conclusion cannot be reached from this. Interestingly, the biofilm formed at 37°C on top of HEp-2 cells had a more significant reduction from DNase treatment than either the biofilm formed at 22°C or 37°C on abiotic surfaces. eDNA production might be further regulated by factors not related to heat-shock, but instead environmental and/or host signals. It is important to note however, that the biofilms formed on top of the cells were in RPMI media supplemented with FBS (compared to the abiotic biofilms formed in HBSS), which could contribute to a different biofilm and resulting DNase treatment efficacy.

Another possible link of eDNA to infection is a recent report with *M*. *tuberculosis* producing extracellular DNA during intracellular infection that exits the perforated phagosome and stimulates the cytosolic surveillance pathway to promote infection [53]. The authors found that the extracellular DNA was mycobacterial in origin and hypothesized that it was secreted, though the mechanism remains elusive. We published a recent manuscript looking at the interaction of MAH biofilms with host surveilling phagocytes, and found that A5 was significantly more stimulatory to phagocytes than MAH 104 [9]. To distinguish if the cells themselves or an acellular component was responsible for this hyper stimulation, we UV-sterilized biofilms prior to infection [9]. The sterilized biofilm elicited an almost identical effect on the phagocytes. Similarly, the acellular supernatants of MAH A5 and 104 biofilms were cultured with phagocytes, and A5 (but not 104) elicited a large response [9]. It is well known that CpG bacterial DNA is a substrate for toll-like receptor 9 in host phagocytes and other cells. When combining those phagocyte stimulation results with the findings of this current report, we speculate that the eDNA could, at least partly, explain the uniquely high TNF-α production elicited from MAH A5. The contribution, if any, of MAH eDNA to virulence still needs to be investigated.

The significant effect of DNase I on the biofilms was intriguing. Biofilm-associated infections with MAH, especially of the respiratory tract, are difficult to treat clinically. We recently published a report establishing the efficacy of a novel inhaled liposomal amikacin for inhalation [54]. Topical therapy delivered by liposomes was effective at reducing the MAH burden in the respiratory tract of mice. It would be worthwhile to investigate the added benefit of possibly combining this topical therapy with an inhaled DNase adjuvant, which is already an FDA approved as an inhaler for cystic fibrosis patients (Pulmozyme). Reducing the tolerance of MAH biofilms *in vivo*, as we demonstrated with DNase *in vitro* in this study, could create more effective treatment options clinically.
